# Supplementary material for: Comprehensive Annotation and Functional Exploration of MicroRNAs in Lettuce
Source: Front Plant Sci. 2021 Dec 24;12:781836. doi: 10.3389/fpls.2021.781836 (PMC8739914; doi:10.3389/fpls.2021.781836)
Supplement: Supplementary file 3 [file Data_Sheet_9.ZIP › GSM2230751.plot/Lsa-miR408_Lsat_1_v5_gn_1_65280.1_452_TPlot.pdf]

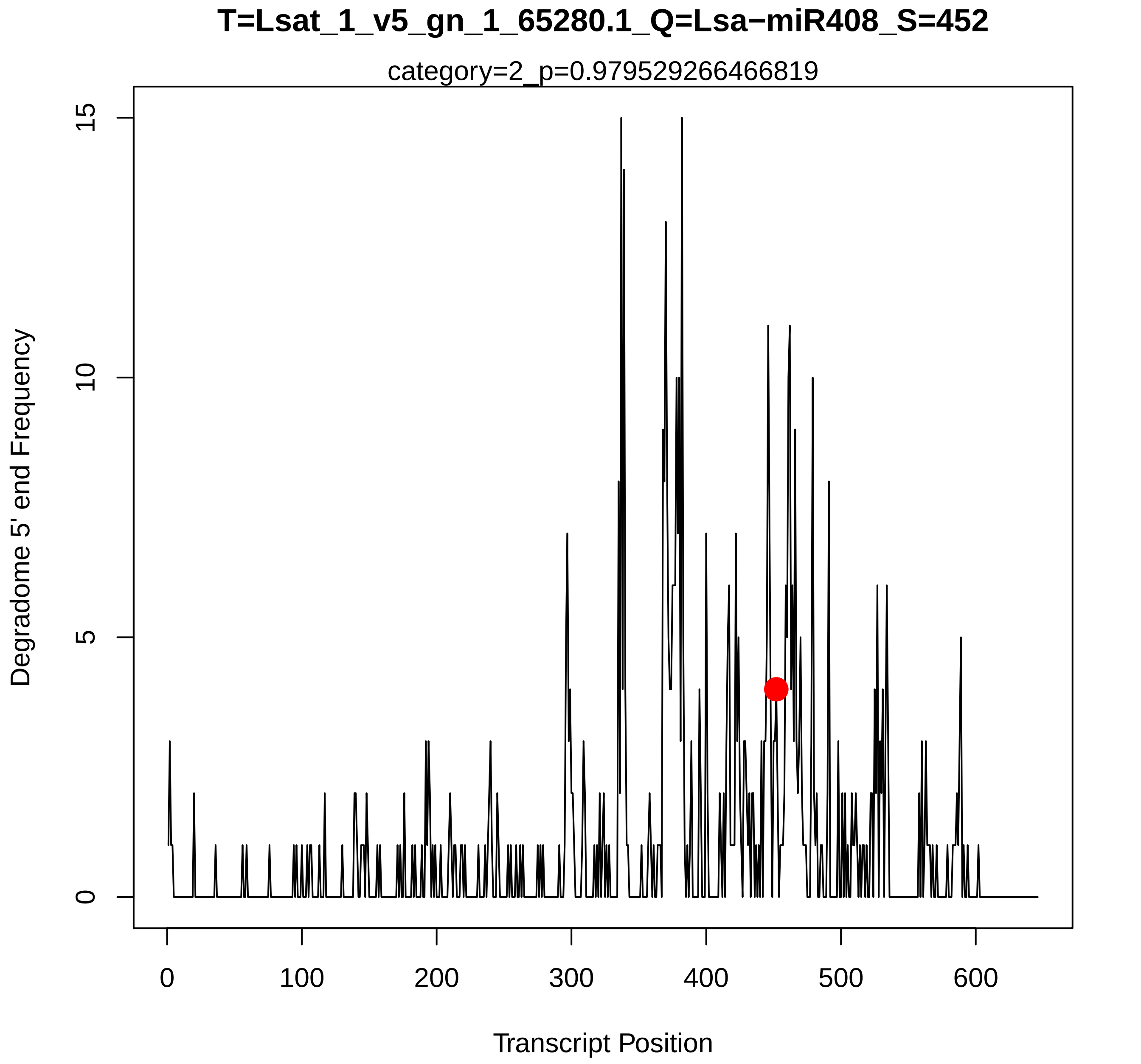
T=Lsat_1_v5_gn_1_65280.1_Q=Lsa−miR408_S=452
category=2_p=0.979529266466819
Degradome 5' end Frequency
0
5
10
15
0
100
200
300
400
500
600
Transcript Position
